# Prevalence and associating factors of long COVID in pediatric patients during the Delta and the Omicron variants

**DOI:** 10.3389/fped.2023.1127582

**Published:** 2023-05-24

**Authors:** Tananya Lokanuwatsatien, Araya Satdhabudha, Auchara Tangsathapornpong, Pornumpa Bunjoungmanee, Phakatip Sinlapamongkolkul, Chanapai Chaiyakulsil, Paskorn Sritipsukho, Pichaya Tantiyavarong

**Affiliations:** ^1^ Department of Pediatrics, Thammasat University Hospital, Thammasat University, Pathum Thani, Thailand; ^2^ Department of Pediatrics, Faculty of Medicine, Thammasat University, Pathum Thani, Thailand; ^3^ Center of Excellence in Applied Epidemiology, Thammasat University, Pathum Thani, Thailand; ^4^ Department of Clinical Epidemiology, Faculty of Medicine, Thammasat University, Pathum Thani, Thailand

**Keywords:** long COVID, SARS-CoV-2, COVID-19, Delta variant, Omicron variant

## Abstract

**Introduction:**

The number of pediatric COVID-19 infections is increasing; however, the data on long COVID conditions in children is still limited. Our study aimed to find the prevalence of long COVID in children during the Delta and Omicron waves, as well as associated factors.

**Methods:**

A single-center prospective cohort study was conducted. We included 802 RT-PCR-confirmed COVID-19 pediatric patients in the Delta and Omicron periods. Long COVID was defined as having symptoms for ≥3 months after infection. Parents and/or patients were interviewed by phone. Multivariable logistic regression was performed to find associated factors with long COVID.

**Results:**

The overall prevalence of long COVID was 30.2%. The Delta period had more prevalence than the Omicron (36.3% vs. 23.9%). Common symptoms for patients 0–3 years’ old were loss of appetite, rhinorrhea, and nasal congestion. Conversely, patients 3–18 years’ old had hair loss, dyspnea on exertion, rhinorrhea, and nasal congestion. However, there was no significant negative impact on daily life. Most symptoms improved after a 6-month follow-up. Factors associated with long COVID-19 conditions were infection during the Omicron period (adjusted OR: 0.54; 95% CI: 0.39–0.74, *P* < 0.001), fever (adjusted OR: 1.49, 95% CI: 1.01–2.20, *P* = 0.04) and rhinorrhea (adjusted OR: 1.47, 95% CI: 1.06–2.02, *P *= 0.02).

**Conclusion:**

Infection during the Omicron wave has a lower prevalence of long COVID. The prognosis is often favorable, and most symptoms gradually become less. However, pediatricians may schedule appointments to surveil long COVID in children with fever or rhinorrhea as an initial symptom.

## Introduction

Since mid-December 2019, a novel coronavirus infection, first detected in Wuhan, China, has rapidly spread throughout the world (1). The infection was first recognized as severe pneumonia with acute respiratory disease syndrome (ARDS), further delineated to be severe acute respiratory syndrome coronavirus 2 (SARS-CoV-2) infection, and now commonly known as COVID-19. The disease's manifestations vary, ranging from asymptomatic seroconversion to critical illness or death.

During the early outbreak, infection prevalence in children was low, and symptoms were usually mild or asymptomatic (2). One year after, new variants emerged and spread rapidly. The Alpha and Delta variants were associated with more pediatric cases, increasing concerns that children would be more vulnerable and sicker (3, 4). Even with the recent Omicron variant showing fewer symptoms, worries have persisted among parents regarding the possibility of acute illness as well as long-term sequelae in their children (5).

Long COVID is an umbrella term that encompasses physical and mental health consequences experienced by patients for four or more weeks following acute COVID-19 illness (6). Long COVID may have significant health and quality of life consequences, regardless of the initial disease severity. The prevalence and symptoms of long COVID in the pediatric population has varied widely among countries and age groups. For example, in Latvia, 51% of outpatients aged ≤18 years were diagnosed with long COVID (7). In the UK, 12.9% of children aged from 2 to 11 and 14.5% of those 12–16 had symptoms five weeks after the initial infection (8). In Australia, only 8% of children had post-acute COVID-19 symptoms (2).

To date, information about long COVID in children remains scarce and inconsistent (7, 9). To describe long-term outcomes in this population comprehensively, including assessing physical and mental health outcomes, telehealth has become essential in screening and follow-up. To our knowledge, no study has compared symptoms of long COVID in pediatric populations between different viral variants; very few studies have mentioned the factors associated with these symptoms. Thus, we created this study to determine the prevalence, clinical characteristics, and associated factors of long COVID by using phone interviews, comparing the Delta and Omicron variant waves. Our data may aid in early detection with appropriate management and provide essential information to overcome parental concerns.

## Materials and methods

### Study design

We conducted a single-center study, which aimed to evaluate the long COVID in children. All COVID-19 patients were <18 years of age and treated at the Thammasat University Hospital. We included first-infection patients with initial infections from September 2021 to March 2022. Diagnosis was performed using nasopharyngeal RT-PCR tests. Based on data from the Thai Government and Ministry of Public Health (MOPH), cases between July 2021 to December 2021 were classified as the Delta dominant period, and from January 2022, it has been considered as the Omicron dominant period (10). The report of SARS-CoV-2 variant surveillance from the Department of Medical Science, MOPH showed the Delta variants of 88%–100% from September to the mid-half of December 2021. Then, the proportion of Omicron variants rapidly increased, accounting for 94%–100% after the mid-half of January 2022 (11), which was consistent with our variant testing among 363 adult and pediatric cases in the study period (Omicron 97.8%: sublineages BA.1 64.8% and BA.2 35.2%). The exclusion criteria were (1) patient mortality or loss of follow-up within three months after infection, (2) unable to communicate in the Thai language, (3) disinclined to consent by phone, and (4) unable to be contacted by phone. This protocol was approved by the Human Research Ethics Committee of Thammasat University No 1 (Faculty of Medicine): MTU-EC-PE-1-326/64.

There were two data sources in our study. First, the baseline characteristics data of included pediatric patients such as age, gender, body weight, comorbidities, infection source, presenting symptoms, as well as severity and treatment data during patient acute illness, were retrieved from the cohort of all patients under investigation (PUI) criteria for COVID-19 at Thammasat University Hospital. Disease severity classification was based on the National Institute of Health (NIH): asymptomatic, mild, moderate, severe, and critically ill (12). All patients were treated with standard protocols according to recommendations from the Department of Medical Service, MOPH (13). Anti-viral therapy was not used for asymptomatic patients. For mild symptoms with comorbidity, favipiravir was prescribed for five days. For moderate symptoms to critical illness, remdesivir and/or systemic steroids were indicated. We also verified individual vaccine status before getting COVID-19 infection using the vaccine database of the MOPH immunization center. Fully vaccinated was defined as at least two-dose regimen according to the Thai Government recommendation.

Second, post-COVID-19 follow-up symptoms of pediatric patients were collected prospectively at three and six months after infection. Informed consent was requested from parents as well as children older than 7 years. Data were collected through a phone survey instead of in-person outpatient visits to ensure the participants’ comfort and reduce hospital visits during the outbreak of COVID-19. Due to diverse symptoms among ages, we designed two specific patient questionnaires: one for those 0–3 years of age and another for 3–18 years of age. To obtain accurate information, questions were asked by trained research assistants. For infants or young children who could not provide information, parents provided data, but for adolescents, they were encouraged to answer by themselves. For the purposes of our study, we defined long COVID as having symptoms for more than three months after infection onset. At the time of phone survey, an appointment for an outpatient visit was also offered to all participants to evaluate an alternative diagnosis. All data were collected in the online standard platform, Research Electronic Data Capture (REDCap) (14, 15).

## Outcomes

The primary outcome was the prevalence of long COVID, comparing the Delta and Omicron waves. We also followed patient symptoms at six months. Unfortunately, there were very few responses in the Omicron group; we suspect that this occurred because symptoms may have disappeared or not been as severe. It is possible that the increased lack of phone follow-up during this time was because there was a proliferation of scam phone calls in Thailand, and many people would not answer calls from unknown numbers. Another important objective was to explore factors associated with long COVID to help inform the risk of long COVID in children.

## Statistical analyses

Sample size estimation was based on the formula of a single proportion. We expected the prevalence of long COVID in our cohort to be 50%. With the assumption of 5% precision and 95% confidence level, the study needed to include at least 485 pediatric patients.

Continuous data were described by the mean and standard deviation or median and interquartile range, as appropriate. Percentages were given as categorical data. Student *t*-test, Mann-Whitney *U*-test, *χ*^2^ test, or Fisher exact test were used to compare baseline characteristics, acute symptoms, prevalence of long COVID, and long COVID symptoms between Delta and Omicron dominant periods.

We used binary logistic regression to determine factors associated with long COVID symptoms in univariable and multivariable models. Stepwise backward procedure for *P* value <0.05 was used. Additionally, we did sensitivity analyses by excluding vague symptoms, such as rhinitis and congestion, from the long COVID definition and then explored the factor associated with long COVID. To evaluate the vaccine effect on long COVID, we excluded children <5 years old in the analysis, which not included in the national vaccine program at the study period. Statistical significance was determined if *P* value <0.05 (two-sided). All analyses were performed using Stata v.17.0 (StataCorp LLC., Texas, USA).

## Results

In our data, 844 RT-PCR-confirmed COVID-19 children were eligible. We excluded 41 cases due to no telephone response. One patient with neurological comorbidities (porencephaly, spastic cerebral palsy, and epilepsy) passed away from COVID-19 pneumonia and was also excluded. There were 802 patients left who completed the interview for long COVID.

Table 1 shows the characteristics of patients at the time of COVID-19 infection, comparing Delta and Omicron periods. Of 802 patients, 179 (22.3%) were aged 0–3 years and 623 (77.7%) in the 3–18 age group. Chronic lung disease was the predominant comorbidity (1.7%). Fever was found less often in the Delta as compared to the Omicron period (12.6% vs. 26.5%, *P* < 0.001), but rhinorrhea (36.3% vs. 29.4%, *P* = 0.040), anosmia (17.4% vs. 0.3%, *P* < 0.001), tastelessness (7.6% vs. 0%, *P* < 0.001), diarrhea (10.3% vs. 4.8%, *P* = 0.003), and rash (2.9% vs. 0%, *P* < 0.001) were more common. Children were generally found to be asymptomatic or had mild symptoms in both periods. Notably, we only saw critical patients during the Delta period but not Omicron. Admission sites differed between the two waves due to a change in Thai government policy: during the Omicron dominant period, patients were permitted to home or community isolate versus previous mandatory hospital admission during the Delta era. Favipiravir was prescribed more often during the Omicron wave; this is likely because favipiravir was given based on the symptom of fever, which presented more often during this time period. The proportion of fully vaccinated cases significantly increased in the Omicron periods (0.2% vs. 25.1%, *P* < 0.001). The common initial regimens were two-doses of BNT162b2 (59 patients), CoronaVac or BBIBP-CorV (22 patients), and ChAdOx1 (10 patients).

**Table 1:** Baseline characteristics of patients.

Characteristics^a^^,^^b^	Total (*N* = 802)	Delta (*N* = 408)	Omicron (*N* = 394)	*P* value
Male sex – no (%)	450 (56.2)	231 (56.8)	219 (55.6)	0.74
**Age–no (%)**				
0–3 year	179 (22.3)	68 (16.7)	111 (28.2)	<0.001
3–18 year	623 (77.7)	340 (83.3)	283 (71.8)	
Body weight – kg	34.2 ± 21.7	35.9 ± 21.5	32.4 ± 21.8	0.021
Height – cm	128.3 ± 32.9	130.2 ± 32.1	126.3 ± 33.7	0.11
**Comorbidities – no (%)**				
Chronic lung disease	14 (1.7)	6 (1.5)	8 (2.0)	0.60
Heart disease	5 (0.6)	1 (0.2)	4 (1.0)	0.21
Diabetes	1 (0.1)	0 (0.0)	1 (0.3)	0.49
Neurologic disease	1 (0.1)	1 (0.2)	0 (0)	1.00
Chronic kidney disease	3 (0.4)	2 (0.5)	1 (0.3)	1.00
Cancer	2 (0.2)	1 (0.2)	1 (0.3)	1.00
**Source of infection – no (%)**				
Household contact	290 (36.7)	136 (33.9)	154 (39.5)	0.022
School	19 (2.4)	5 (1.2)	14 (3.6)	
Hospital	2 (0.3)	0 (0.0)	2 (0.5)	
Cluster/Community	20 (2.5)	12 (3.0)	8 (2.1)	
Other	460 (58.2)	248 (61.8)	212 (54.4)	
**Acute symptom – *n* (%)**				
Fever (≥37.5°C)	155 (19.4)	51 (12.6)	104 (26.5)	<0.001
Cough	412 (51.4)	214 (52.5)	198 (50.3)	0.53
Rhinorrhea	264 (32.9)	148 (36.3)	116 (29.4)	0.040
Anosmia	72 (9.0)	71 (17.4)	1 (0.3)	<0.001
Tasteless	31 (3.9)	31 (7.6)	0 (0.0)	<0.001
Dyspnea/Tachypnea	24 (3.0)	14 (3.4)	10 (2.5)	0.46
Diarrhea	61 (7.6)	42 (10.3)	19 (4.8)	0.003
Headache/Vertigo	84 (10.5)	40 (9.8)	44 (11.2)	0.53
Myalgia/Arthralgia	39 (4.9)	18 (4.4)	21 (5.3)	0.55
Rash	12 (1.5)	12 (2.9)	0 (0.0)	<0.001
Sore throat	194 (24.2)	81 (19.9)	113 (28.7)	0.004
**Peak severity – *n* (%)**				
Asymptomatic	131 (16.5)	86 (21.3)	45 (11.5)	0.002
Mild	608 (76.5)	286 (70.8)	322 (82.4)	
Moderate	48 (6.0)	27 (6.7)	21 (5.4)	
Severe	6 (0.8)	3 (0.7)	3 (0.8)	
Critical	2 (0.3)	2 (0.5)	0 (0)	
Moderate to severe symptoms	56 (7.0)	32 (7.9)	24 (6.1)	0.33
**Admission site – *n* (%)**				
Home isolation	108 (14.7)	36 (8.9)	72 (21.8)	<0.001
Field hospital	132 (17.9)	129 (31.8)	3 (0.9)	
Hospital	421 (57.1)	241 (59.4)	180 (54.4)	
Referral	76 (10.3)	0 (0.0)	76 (23.0)	
**Medication – no (%)**				
Favipiravir	400 (50.1)	118 (28.9)	282 (72.1)	<0.001
Remdesivir	2 (0.3)	2 (0.5)	0 (0.0)	0.17
Dexamethasone	6 (0.8)	5 (1.2)	1 (0.3)	0.22
**Respiratory equipment – *n* (%)**				
Cannulas/Mask	4 (44)	3 (60)	1 (25)	0.079
HHFNC	3 (33)	0 (0)	3 (75)	
NIPPV	0 (0)	0 (0)	0 (0)	
Ventilator	2 (22)	2 (40)	0 (0)	
**Vaccination status** ^c^				
No vaccination	662 (82.5)	379 (97.3)	265 (67.3)	<0.001
One dose	40 (5.0)	10 (2.5)	30 (7.6)	
Two doses	84 (10.5)	1 (0.2)	83 (21.1)	
Three doses	15 (1.9)	0 (0)	15 (3.8)	
Four doses	1 (0.1)	0 (0)	1 (0.3)	
Fully vaccinated^c^	100 (12.5)	1 (0.2)	99 (25.1)	<0.001

HHFNC, high flow oxygen therapy.

^a^ Data are presented as mean ± standard deviation for continuous variables and number (percent) for categorical variables. Percentages may not total 100 due to rounding.

^b^ Non-normal distributed variables are presented as median [interquartile range].

^c^ Fully vaccinated was defined as at least two-dose regimen according to the Thai Government recommendation.

**Figure 1:**
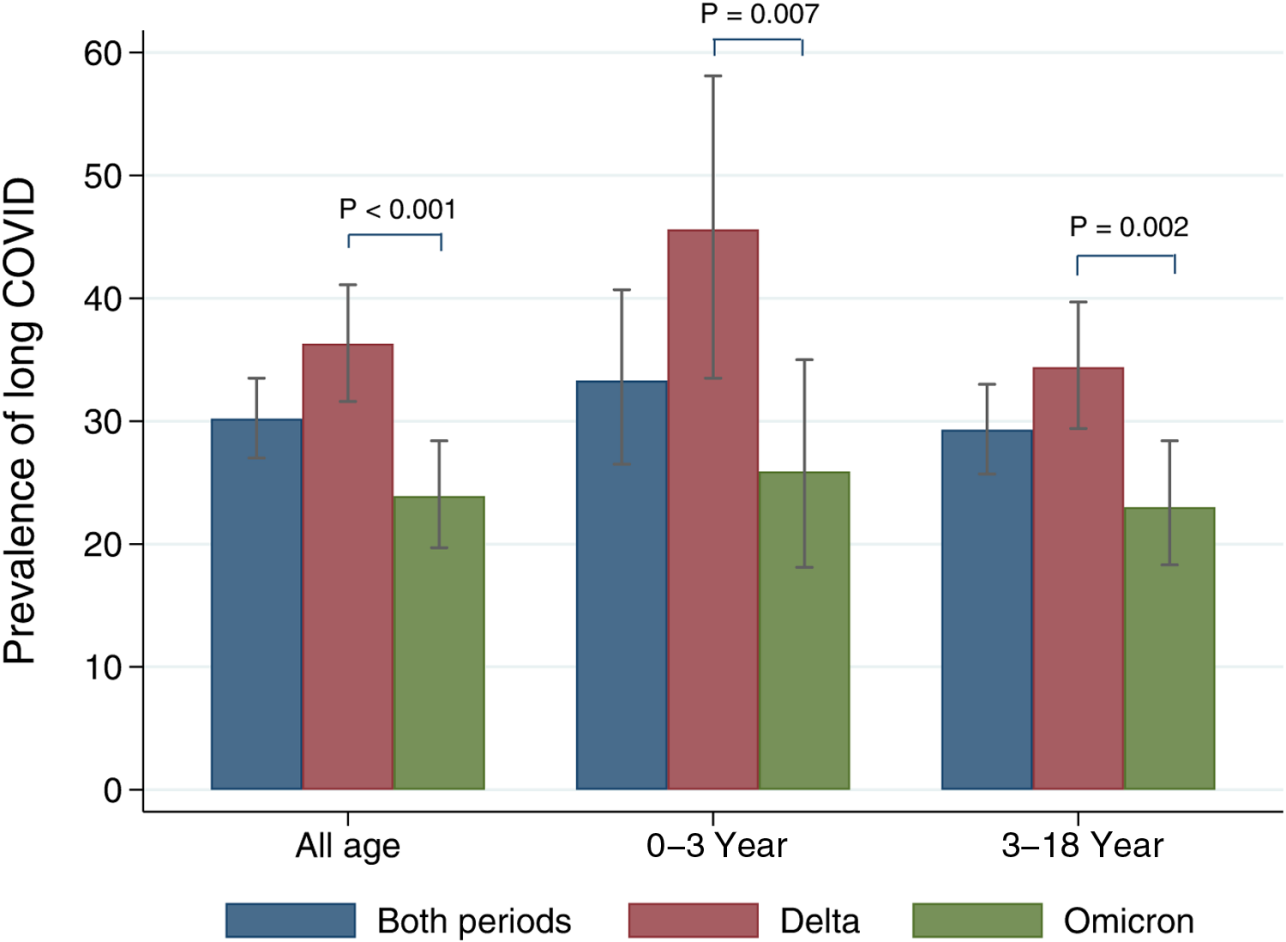
Prevalence of long COVID stratified by age groups and COVID-19 variant periods. The grey bar indicates 95% confidence intervals.

The overall prevalence of long COVID was 30.2% (95% CI: 27.0–33.5) (Figure 1). The Delta period had more prevalence than the Omicron (36.3% vs. 23.9%). Prevalence in all age, 0–3 year, and 3–18 year groups were significantly predominant during the Delta period. Patients 0–3 years’ old had more symptoms than those aged 3–18 years (33.3% vs. 29.3%). Tables 2, 3 show long COVID symptoms in the two different age cohorts. In both waves, three months after COVID-19 infection, common symptoms for the younger group were loss of appetite, rhinorrhea, and nasal congestion. Nasal congestion was found more often during Delta (14.7% vs. 0.9%, *P <* 0.001). On the contrary, children aged 3–18 years during Delta had more anosmia (1.8% vs. 0%, *P* = 0.03), weight loss (2.6% vs. 1.4, *P *= 0.005), and hair loss (10.6% vs. 3.9%, *P* = 0.002) when compared to the Omicron period. Moreover, inattention was more common during Omicron (4.6% vs. 0.3%, *P* = 0.001). However, there was no difference impact on daily life of long COVID symptoms between infection periods in both age groups (Tables 4 and 5). Follow-up by telehealth six months after initial infection showed that symptoms improved over time (Supplementary Tables S1 and S2).

**Table 2:** Long COVID symptoms in 0–3-year-old patients at 3 months^a^.

Symptoms	Total (*N* = 179)	Delta (*N* = 68)	Omicron (*N* = 111)	*P* value
**Non-specific symptom**				
Fever	6 (3.4)	3 (4.4)	3 (2.7)	0.67
Malaise	2 (1.1)	0 (0.0)	2 (1.8)	0.53
Loss of appetite	14 (7.8)	3 (4.4)	11 (9.9)	0.25
Weight loss	2 (1.1)	2 (2.9)	0 (0.0)	0.14
**Eye**				
Red eye	0 (0)	0 (0)	0 (0)	NA
**Respiratory**				
Cough	9 (5.0)	4 (5.9)	5 (4.5)	0.73
Rhinorrhea	14 (7.8)	8 (11.8)	6 (5.4)	0.15
Congestion	11 (6.1)	10 (14.7)	1 (0.9)	<0.001
Dyspnea at rest	5 (2.8)	4 (5.9)	1 (0.9)	0.07
Dyspnea on exertion	4 (2.2)	2 (2.9)	2 (1.8)	0.64
**GI**				
Diarrhea	4 (2.2)	3 (4.4)	1 (0.9)	0.15
Vomiting	1 (0.6)	1 (1.5)	0 (0.0)	0.38
Constipation	9 (5.0)	4 (5.9)	5 (4.5)	0.73
**CNS**				
Abnormal balance	0 (0.0)	0 (0.0)	0 (0.0)	NA
Drowsiness	0 (0.0)	0 (0.0)	0 (0.0)	NA
**Skin**				
Rash	9 (5.0)	3 (4.4)	6 (5.4)	1.00
Excessive sweat	3 (1.7)	3 (4.4)	0 (0.0)	0.053
Hair loss	5 (2.8)	4 (5.9)	1 (0.9)	0.07

^a^ Data are presented as number and percentages. Percentages may not total 100 due to rounding.

**Table 3:** Long COVID symptoms in 3–18-year-old patients at 3 months^a^.

Symptoms	Total (*N* = 623)	Delta (*N* = 340)	Omicron (*N* = 283)	*P* value
**Non-specific symptom**				
Fever	3 (0.5)	1 (0.3)	2 (0.7)	0.59
Sore throat	4 (0.6)	1 (0.3)	3 (1.1)	0.33
Anosmia	6 (1.0)	6 (1.8)	0 (0.0)	0.034
Ageusia	2 (0.3)	2 (0.6)	0 (0.0)	0.50
Fatigue/Malaise	4 (0.6)	2 (0.6)	2 (0.7)	1.00
Loss of appetite	13 (2.1)	8 (2.4)	5 (1.8)	0.61
Weight loss	9 (1.4)	9 (2.6)	0 (0.0)	0.005
**Eye**				
Red eye	1 (0.2)	0 (0.0)	1 (0.4)	0.45
Dry eye	3 (0.5)	2 (0.6)	1 (0.4)	1.00
**Respiratory**				
Cough	16 (2.6)	9 (2.6)	7 (2.5)	0.89
Rhinorrhea	24 (3.9)	15 (4.4)	9 (3.2)	0.43
Congestion	24 (3.9)	14 (4.1)	10 (3.5)	0.70
Dyspnea at rest	3 (0.5)	2 (0.6)	1 (0.4)	1.00
Dyspnea on exertion	27 (4.3)	13 (3.8)	14 (4.9)	0.50
Shortness of breath	7 (1.1)	3 (0.9)	4 (1.4)	0.71
**CVS**				
Chest pain	3 (0.5)	2 (0.6)	1 (0.4)	1.00
Palpitation	2 (0.3)	1 (0.3)	1 (0.4)	1.00
Faint/Syncope	6 (1.0)	3 (0.9)	3 (1.1)	1.00
**GI**				
Diarrhea	10 (1.6)	7 (2.1)	3 (1.1)	0.36
Nausea	0 (0)	0 (0)	0 (0)	NA
Vomiting	2 (0.3)	2 (0.6)	0 (0.0)	0.50
Abdominal pain	7 (1.1)	4 (1.2)	3 (1.1)	1.00
Constipation	12 (1.9)	8 (2.4)	4 (1.4)	0.56
**Growth & Development**				
Inattention	14 (2.2)	1 (0.3)	13 (4.6)	<0.001
Hyperactive	7 (1.1)	4 (1.2)	3 (1.1)	1.00
**CNS**				
Headache	9 (1.4)	6 (1.8)	3 (1.1)	0.52
Vertigo	7 (1.1)	4 (1.2)	3 (1.1)	1.00
Numbness	9 (1.4)	6 (1.8)	3 (1.1)	0.52
Abnormal balance	0 (0.0)	0 (0.0)	0 (0.0)	NA
Tremor	1 (0.2)	1 (0.3)	0 (0.0)	1.00
**Mood**				
Stress	4 (0.6)	2 (0.6)	2 (0.7)	1.00
Sad	2 (0.3)	1 (0.3)	1 (0.4)	1.00
Anxiety	2 (0.3)	1 (0.3)	1 (0.4)	1.00
**Skin**				
Rash	8 (1.3)	5 (1.5)	3 (1.1)	0.73
Excessive sweat	8 (1.3)	2 (0.6)	6 (2.1)	015
Hair loss	47 (7.5)	36 (10.6)	11 (3.9)	0.002
**Rheumatic symptom**				
Myalgia	20 (3.2)	12 (3.5)	8 (2.8)	0.62
Joint pain	12 (1.9)	7 (2.1)	5 (1.8)	1.00

^a^ Data are presented as number and percentages. Percentages may not total 100 due to rounding.

**Table 4:** Negative impact on daily life of long COVID symptoms in 0–3-year-old patients at 3 months.

Domains^a^	Total (*N* = 179)	Delta (*N* = 68)	Omicron (*N* = 111)	*P* value
Eat	6 (3.4)	1 (1.5)	5 (4.5)	0.41
Sleep	4 (2.2)	2 (2.9)	2 (1.8)	0.64
Play	1 (0.6)	1 (1.5)	0 (0)	0.38

^a^ Data are presented as number and percentages. Percentages may not total 100 due to rounding.

**Table 5:** Negative impact on daily life of long COVID symptoms in 3–18-year-old patients at 3 months.

Domains^a^	Total (*N* = 623)	Delta (*N* = 340)	Omicron (*N* = 283)	*P* value
Eat	11 (1.8)	8 (2.4)	3 (1.1)	0.36
Sleep	6 (1.0)	3 (0.9)	3 (1.1)	1.00
Daily activities	1 (0.2)	1 (0.3)	0 (0.0)	1.00
Learning	5 (0.8)	3 (0.9)	2 (0.7)	1.00
Play or exercise	6 (1.0)	2 (0.6)	4 (1.4)	0.42

^a^ Data are presented as number and percentages. Percentages may not total 100 due to rounding.

Factors associated with long COVID-19 symptoms are displayed in Table 6. In univariable analyses, infections during the Omicron era were significantly associated with a reduction in the risk of developing long COVID-19 (crude odd ratio (OR) 0.55; 95% CI: 0.40–0.75, *P* < 0.001). During both waves, other significant factors associated with developing long COVID were having moderate to severe symptoms (crude OR: 1.81; 95% CI: 1.05–3.16, *P* = 0.03), rhinorrhea (crude OR: 1.5; 95% CI: 1.09–2.05, *P* = 0.01), diarrhea (crude OR: 1.94; 95% CI: 1.14–3.30, *P* = 0.01), and rash (crude OR: 3.31; 95% CI: 1.04–10.52, *P* = 0.04). After performing multivariable analyses with stepwise backward elimination for both eras, significant factors associated with long COVID-19 conditions were COVID-19 infection during the Omicron period (adjusted OR: 0.54; 95% CI: 0.39–0.74, *P* < 0.001), fever (adjusted OR: 1.49, 95% CI: 1.01–2.20, *P* = 0.04) and rhinorrhea (adjusted OR: 1.47, 95% CI: 1.06–2.02, *P* = 0.02). The sensitivity analyses excluding vague symptoms showed that the Omicron period was the only robust predictor associated with less occurrence of long COVID compared to the Delta period (Supplementary Table S3). Additionally, vaccination was not associated with occurrence of long COVID-19 in ≥5-year-old patients (*N* = 559) (crude OR: 0.94; 95% CI: 0.58–1.53, *P* = 0.81).

**Table 6:** Factors associated with long COVID symptoms.

Factor	Univariable analysis^b^			Multivariable analysis^c^		
	OR	95% CI	*P* value	OR	95% CI	*P* value
Omicron	0.55	0.40–0.75	<0.001	0.54	0.39–0.74	<0.001
Female	1.10	0.81–1.49	0.54			
Age 3–18 years	0.83	0.58–1.18	0.30			
Any comorbidity^a^	1.09	0.46–2.57	0.84			
≥Moderate symptom	1.81	1.05–3.16	0.03			
Favipiravir	0.86	0.64–1.17	0.34			
Remdesivir	2.31	0.14–37.0	0.56			
Systemic steroid	0.49	0.05–3.94	0.48			
Fever (≥37.5°C)	1.27	0.87–1.84	0.21	1.49	1.01–2.20	0.043
Cough	1.26	0.93–1.71	0.14			
Rhinorrhea	1.50	1.09–2.05	0.01	1.47	1.06–2.02	0.019
Anosmia	1.17	0.70–1.97	0.54			
Tasteless	1.11	0.51–2.39	0.80			
Dyspnea	2.00	0.88–4.54	0.10			
Diarrhea	1.94	1.14–3.30	0.01			
Myalgia	1.48	0.76–2.87	0.25			
Rash	3.31	1.04–10.52	0.04			
Sore throat	1.19	0.84–1.69	0.33			

OR, odds ratio.

^a^ Any comorbidity was one or more of the followings: chronic lung disease, cardiovascular disease, diabetes mellitus, neurological disease, chronic kidney disease, cancer.

^b^ In univariable analysis, simple binary logistic regression was used.

^c^ In multivariable analysis, adjusted binary logistic regression was performed using stepwise regression (*P* value < 0.05).

## Discussion

This study investigated the prevalence of long COVID in pediatric patients and its associated factors: overall prevalence was 30.2%. Long COVID was significantly higher during the Delta era in all age groups, and it occurred more often in patients aged 0–3- years versus those 3–18 years. Infection during the Omicron period showed a lower prevalence of long COVID. Furthermore, fever and rhinorrhea at the time of acute illness were associated with long COVID.

Previous studies reported a variety of prevalence for long COVID and with different symptoms. A systematic review and meta-analysis in children and adolescents showed a prevalence of 25% (95% CI: 18–33%) (16). The most dominant clinical symptoms were mood symptoms (16.5%), fatigue (9.7%), sleep disorder (8.4%), headache (7.8%), and respiratory symptoms (7.6%). The authors mentioned high heterogeneity and high probabilities of bias due to a lack of standardized definitions, recall, nonresponse, and a difference in follow-up and age groups. Our study found a prevalence of 30.2% for long COVID, a similar range to previous reports. However, our main symptoms were respiratory, not mood or fatigue which are quite subjective to monitor. Most of the studies (16 out of 21) in the systematic review and meta-analysis included a large number of adolescent cases. Older children can typically describe their symptoms well, unlike young ones who cannot completely verbalize their symptoms. Thus, our common symptoms differed. We also confirmed that the Omicron variant had a lower prevalence than Delta, consistent with findings in adult studies (17).

Associated factors in this study were COVID-19 infection in the Omicron period, clinical symptoms of fever, and rhinorrhea. Infection during the Omicron period was a robust predictor associated with less frequency of long COVID in our sensitivity analyses. Similarly, a recent study from a pediatric post-COVID clinic in Italy showed that the Omicron variant had a lower risk of long COVID than the previous variants (Wild, Alfa, and Delta variants) (18). Another study revealed that the risk factors for persistent symptoms were when the children were older and had a history of allergic disease (19). The symptoms of rhinorrhea may be a part of the allergic disease that was unchecked before the COVID-19 infection. One-fourth of them were diagnosed with allergic rhinitis at our hospital. Others were checked at their own health care provider. Patients with allergic disease might have an increased risk of long COVID, resulting from T helper 2 immunological response and may be linked with mast cell activation (20, 21).

While there are fewer cases of long COVID at this time, pediatricians should schedule appointments to evaluate the risk of long COVID, particularly if patients present with fever or rhinorrhea as these patients may actually have an allergic disease. Nonetheless, the symptom of long COVID in children is not severe and has good prognosis, so parents should not be as concerned. Similar to the data from the nationwide cohort studies, long COVID in children is not highly prevalent and mainly short of duration (22, 23).

COVID-19 vaccination before infection seems to reduce long COVID in adult patients. Preliminary evidence suggested that two doses can reduce symptoms better than one dose (24). A recent systematic review (25), which searched studies from January 2020 to August 2022, included 16 observational studies in 614,392 patients. Ten studies showed a significant reduction in long COVID incidence; however, the authors did not conduct meta-analyses due to high heterogeneity. The result should be interpreted for early variants because most studies included patients in the pre-Omicron era. Unlike the adult data, our study could not demonstrate the association between receiving vaccine before infection and long COVID occurrence. One explanation may be a mild degree of long COVID symptoms in children, especially in the Omicron period. Other might be the low-vaccine coverage among the participants during the study period. Thus, we cannot find the positive effect of vaccination.

The strength of this study is its large sample size, which has helped further identify the prevalence, signs, symptoms, and risk factors of long COVID in children, during the Delta and Omicron waves, specifically in Asia. In addition, all COVID-19 cases were confirmed by RT-PCR, and we defined long COVID as having signs and symptoms persisting more than three months after initial infection.

However, our major limitation was the general lack of consensus for any standardized definitions and criteria for the definitive diagnosis of long COVID as this is new phenomenon, leading to difficulty in representing and comparing the true prevalence of the condition. We used phone interviews; thus, performing a physical examination or laboratory investigations to confirm the definitive diagnosis of particular complaints was impossible. Regardless of our efforts to offer an appointment for an in-person visit for all participants, most patients (73%) failed to show up for further appointments because their symptoms improved, as well as fear of contracting more infections from the hospital, which was the real-life situation. Therefore, we were unable to identify the definitive pathogen responsible for the symptoms. Our nonresponsive population may have altered the true prevalence of long COVID in children. Finally, there was no control group in children with similar demographics without COVID-19 infection to help elucidate whether these constellations of symptoms were or were not possibly related to isolation and lockdowns.

## Conclusion

Long COVID in children is common, and the clinical manifestations vary among individuals and involve multiple organ systems. Nonetheless, the prognosis of long COVID is generally good, and the majority of symptoms go better with time. The Omicron variant has a lower chance of developing long COVID. Patients, who present with fever and rhinorrhea in acute COVID-19 infection, should be monitored for long COVID. Further studies are paramount to understanding the prevention, providing appropriate care, and follow-up of long COVID in pediatric patients.

## Data Availability

The raw data supporting the conclusions of this article will be made available by the authors, without undue reservation.
