# Supplementary material for: Prevalence and associating factors of long COVID in pediatric patients during the Delta and the Omicron variants
Source: Front Pediatr. 2023 May 24;11:1127582. doi: 10.3389/fped.2023.1127582 (PMC10244785; doi:10.3389/fped.2023.1127582)
Supplement: Supplementary file 1 [file Table1.docx]

Supplementary Material

**Prevalence and associating factors of long COVID in pediatric patients during the Delta and the Omicron variants**

**Tananya Lokanuwatsatien, Araya Satdhabudha, Auchara Tangsathapornpong, Pornumpa Bunjoungmanee, Pakatip Sinlapamongkolkul, Chanapai Chaiyakulsil, Paskorn Sritipsukho, Pichaya Tantiyavarong^*^**

*** Correspondence:**Pichaya Tantiyavarong

Email: pichaya_t@tu.ac.th

**Supplementary Table 1.** Long COVID symptoms in 0-3-year-old patients at 3 and 6 months in the Delta period.

| Symptoms^a^ | 3 Month  (N = 68) | 6 Month  (N = 68) |
| --- | --- | --- |
| Non-specific symptom |  |  |
| Fever | 3 (4.4) | 0 (0) |
| Malaise | 0 (0.0) | 0 (0) |
| Loss of appetite | 3 (4.4) | 1 (1.5) |
| Weight loss | 2 (2.9) | 2 (2.9) |
| Eye |  |  |
| Red eye | 0 (0) | 0 (0) |
| Respiratory |  |  |
| Cough | 4 (5.9) | 1 (1.5) |
| Rhinorrhea | 8 (11.8) | 4 (5.9) |
| Congestion | 10 (14.7) | 5 (7.4) |
| Dyspnea at rest | 4 (5.9) | 0 (0) |
| Dyspnea on exertion | 2 (2.9) | 1 (1.5) |
| GI |  |  |
| Diarrhea | 3 (4.4) | 2 (2.9) |
| Vomiting | 1 (1.5) | 1 (1.5) |
| Constipation | 4 (5.9) | 4 (5.9) |
| CNS |  |  |
| Abnormal balance | 0 (0) | 0 (0) |
| Drowsiness | 0 (0) | 0 (0) |
| Skin |  |  |
| Rash | 3 (4.4) | 2 (2.9) |
| Excessive sweat | 3 (4.4) | 1 (1.5) |
| Hair loss | 4 (5.9) | 1 (1.5) |

^a^ Data are presented as numbers and percentages. Percentages may not total 100 due to rounding.

Note: Follow-up data at 6 months had a lot of missing because participants did not take a phone calls for an interview.

**Supplementary Table 2.** Long COVID symptoms in 3-18-year-old patients at 3 and 6 months in Delta period.

| Symptoms^a^ | 3 Month  (N = 340) | 6 Month  (N = 332) |
| --- | --- | --- |
| Non-specific symptom |  |  |
| Fever | 1 (0.3) | 2 (0.6) |
| Sore throat | 1 (0.3) | 0 (0) |
| Anosmia | 6 (1.8) | 1 (0.3) |
| Ageusia | 2 (0.6) | 0 (0) |
| Fatigue / Malaise | 2 (0.6) | 2 (0.6) |
| Loss of appetite | 8 (2.4) | 6 (1.8) |
| Weight loss | 9 (2.6) | 7 (2.1) |
| Eye |  |  |
| Red eye | 0 (0.0) | 0 (0) |
| Dry eye | 2 (0.6) | 0 (0) |
| Respiratory |  |  |
| Cough | 9 (2.6) | 10 (3.0) |
| Rhinorrhea | 15 (4.4) | 5 (1.5) |
| Congestion | 14 (4.1) | 7 (2.1) |
| Dyspnea at rest | 2 (0.6) | 2 (0.6) |
| Dyspnea on exertion | 13 (3.8) | 9 (2.7) |
| Shortness of breath | 3 (0.9) | 4 (1.2) |
| CVS |  |  |
| Chest pain | 2 (0.6) | 2 (0.6) |
| Palpitation | 1 (0.3) | 1 (0.3) |
| Faint / Syncope | 3 (0.9) | 2 (0.6) |
| GI |  |  |
| Diarrhea | 7 (2.1) | 1 (0.3) |
| Nausea | 0 (0) | 0 (0) |
| Vomiting | 2 (0.6) | 0 (0) |
| Abdominal pain | 4 (1.2) | 1 (0.3) |
| Constipation | 8 (2.4) | 2 (0.6) |
| Growth & Development |  |  |
| Inattention | 1 (0.3) | 0 (0) |
| Hyperactive | 4 (1.2) | 2 (0.6) |
| CNS |  |  |
| Headache | 6 (1.8) | 2 (0.6) |
| Vertigo | 4 (1.2) | 1 (0.3) |
| Numbness | 6 (1.8) | 3 (0.9) |
| Abnormal balance | 0 (0) | 0 (0) |
| Tremor | 1 (0.3) | 0 (0) |
| Mood |  |  |
| Stress | 2 (0.6) | 2 (0.6) |
| Sad | 1 (0.3) | 0 (0) |
| Anxiety | 1 (0.3) | 1 (0.3) |
| Skin |  |  |
| Rash | 5 (1.5) | 0 (0) |
| Excessive sweat | 2 (0.6) | 0 (0) |
| Hair loss | 36 (10.6) | 9 (2.7) |
| Rheumatic symptom |  |  |
| Myalgia | 12 (3.5) | 5 (1.5) |
| Joint pain | 7 (2.1) | 2 (0.6) |

^a^ Data are presented as numbers and percentages. Percentages may not total 100 due to rounding.

Note: Follow-up data at 6 months had a lot of missing data because participants did not answer phone calls for an interview.

**Supplementary Table 3.** Sensitivity analyses to explore factors associated with long COVID (excluding vague respiratory symptoms).

| Factor | Univariable analysis^b^ | | | Multivariable analysis^c^ | | |
| --- | --- | --- | --- | --- | --- | --- |
|  | OR | 95% CI | P value | OR | 95% CI | P value |
| Omicron | 0.57 | 0.42-0.79 | 0.001 | 0.54 | 0.39-0.75 | <0.001 |
| Female | 1.18 | 0.87-1.62 | 0.29 |  |  |  |
| Age 3-18 years | 0.96 | 0.66-1.39 | 0.82 |  |  |  |
| Any comorbidity^a^ | 1.24 | 0.53-2.91 | 0.62 |  |  |  |
| ≥ Moderate symptom | 1.77 | 1.01-3.11 | 0.045 | 2.33 | 1.28-4.22 | 0.005 |
| Favipiravir | 0.80 | 0.59-1.10 | 0.17 |  |  |  |
| Remdesivir | omitted^d^ |  |  |  |  |  |
| Systemic steroid | omitted^d^ |  |  |  |  |  |
| Fever (≥ 37.5^o^C) | 1.12 | 0.76-1.65 | 0.55 |  |  |  |
| Cough | 1.25 | 0.92-1.71 | 0.16 |  |  |  |
| Rhinorrhea | 1.30 | 0.94-1.79 | 0.12 |  |  |  |
| Anosmia | 1.25 | 0.74-2.11 | 0.40 |  |  |  |
| Tasteless | 1.26 | 0.58-2.71 | 0.56 |  |  |  |
| Dyspnea | 1.32 | 0.56-3.12 | 0.53 |  |  |  |
| Diarrhea | 1.78 | 1.04-3.05 | 0.04 |  |  |  |
| Myalgia | 1.49 | 0.76-2.93 | 0.24 |  |  |  |
| Rash | 1.31 | 0.39-4.40 | 0.66 |  |  |  |
| Sore throat | 1.39 | 0.97-1.97 | 0.07 | 1.56 | 1.09-2.25 | 0.015 |

^a^ Any comorbidity was one or more of the followings: chronic lung disease, cardiovascular disease, diabetes mellitus, neurological disease, chronic kidney disease, cancer.

^b^ In univariable analysis, simple binary logistic regression was used.

^c^ In multivariable analysis, adjusted binary logistic regression was performed using stepwise regression (P value < 0.05).

^d^ Odds ratio was omitted due to the perfect prediction of long COVID.

Abbreviations: OR, odds ratio.
